# The potential for bridging: HIV status awareness and risky sexual behaviour of injection drug users who have non-injecting permanent partners in Ukraine

**DOI:** 10.7448/IAS.17.1.18825

**Published:** 2014-02-17

**Authors:** Alyona Mazhnaya, Tatiana I Andreeva, Steve Samuels, Jack DeHovitz, Tetyana Salyuk, Louise-Anne McNutt

**Affiliations:** 1 School of public health, National University of Kyiv-Mohyla Academy, Kyiv, Ukraine; 2 School of Public Health, State University of New York at Albany, Albany, NY, USA; 3 Department of Medicine, SUNY-Downstate Medical Center, Brooklyn, NY, USA; 4 Monitoring and Evaluation, International HIV/AIDS Alliance in Ukraine, Kyiv, Ukraine; 5 Institute for Health and Environment, State University of New York at Albany, Rensselaer, NY, USA

**Keywords:** HIV testing, Ukraine, IDU, HIV epidemic generalization, sexual behaviour

## Abstract

**Objective:**

To quantify potential bridging of HIV transmission between the injection drug using subpopulation to the non-injection drug using population through unprotected heterosexual sex.

**Design:**

Secondary analysis of cross-sectional data.

**Methods:**

A sub-sample of participants who reported having a permanent partner who are not injection drug users and have not injected drugs in the past (*N*=1379) was selected from a survey implemented in 26 Ukrainian cities in 2011. This study evaluates the association between consistent condom use and awareness of HIV status as measured by rapid testing during the study (known/unknown HIV+, known/unknown HIV− and undetermined) among a sub-sample of male injection drug users (IDUs) who have a non-injecting permanent partner. Poisson regression, with robust variance estimates, was utilized to identify associations while adjusting for other factors.

**Results:**

Reported consistent condom use varied between 15.5% (unknown HIV−) and 37.5% (known HIV+); average use was 19.3%. In multivariate analysis, males who were aware of their HIV+ status were more likely to report recent consistent condom use compared to those who were unaware of their HIV+ status. This association remains after adjustment for age, region, education level, years of injection, alcohol use, self-reported primary drug use and being an NGO client (prevalence ratio=1.65; 95% CI 1.03–2.64). No such association was found for those who were HIV−.

**Conclusions:**

Our results regarding HIV-positive male IDUs reinforce previous findings that HIV testing and counselling may be an effective means of secondary prevention. Further research is needed to understand how to effectively promote safer sex behaviours for IDUs who are currently HIV−.

## Introduction

According to a recent global report on AIDS, high rates of HIV transmission continue to occur among networks of people who inject drugs and their sexual partners in Eastern Europe [1]. There are many factors that contribute to the on-going problem of HIV among injection drug users (IDUs) in this region and elsewhere. Among them are difficulties in changing sexual behaviours per se, which are often additionally complicated by drug use, and linkages between drug user groups, which differ in HIV prevalence. The bridges between these groups are created by drug use, sex and social networks, and a high prevalence of other sexually transmitted infections that facilitate HIV transmission [2].

The high HIV prevalence among IDUs has the potential to trigger a self-sustaining epidemic which crosses over to non-injection drug using heterosexuals, primarily the non-injecting sex partners [3]. This potential transition from an epidemic mediated by injection drug use mediated epidemic to a heterosexual epidemic has been discussed broadly in the literature, in Ukraine and globally [2–12].

Two defining characteristics of the HIV epidemic in Ukraine today are an increased prevalence and increased number of new registered cases attributed to heterosexual transmission of HIV. However, current prevention programs insufficiently address the particular risks of sexual behaviour among IDUs [2, 13].

In 2012, there were 20,743 new cases of HIV infection registered in Ukraine [14], 51.1% of them were qualified as due to sexual route of transmission, which re-establishes the relevance of “know your epidemic” efforts [15].

For now, the epidemic in Ukraine remains concentrated in high-risk groups of HIV infection such as IDUs, commercial sex workers and men who have sex with men. Yet a key remaining question is the likelihood of epidemic transition from IDUs to their non-injecting partners who may serve as a bridge population for epidemic generalization. Among the most effective strategies for reducing sexual transmission from HIV-infected persons are testing and counselling [16] which promote behaviour change, for example, increased condom use and early initiation of antiretroviral therapy with the expected reduction of viral replication [17]. In addition, pre-exposure prophylaxis has been shown to be effective among serodiscordant heterosexual couples [18, 19]. Because of inadequate access to antiretroviral therapy in Ukraine, testing and counselling are the first line of defence for secondary HIV prevention; however, it is not clear whether this intervention is effective in reducing risky sexual behaviour among those IDUs who have non-injecting sexual partners.

The main objective of this study is to assess the potential for bridging of HIV infection from the injection drug using to the non-injection drug using population. Thus, we investigated the association between consistent condom use and knowledge of one's HIV status among a sub-sample of IDUs who have a non-injecting permanent partner.

## Methods

### Sample

Data used in this study were originally collected as a part of the bi-annual survey “Monitoring the behavior and HIV-infection prevalence among IDUs as a component of second-generation HIV surveillance” implemented in 26 Ukrainian cities in 2011 [20]. The overall goals of the survey were to study the behavioural practices of injection drug use, condom use, HIV testing, level of knowledge about HIV transmission routes, and examine the prevalence of HIV and hepatitis C virus infections among IDUs. Data were gathered using respondent-driven sampling (RDS) techniques to recruit males and females who met the following eligibility criteria: aged at least 14 years upon enrolment; practiced injection drug use in the last 30 days; did not participate in a survey in the last six months; resided, worked, or studied in the polling city; agreed to participate in a survey; and agreed to undergo blood testing for HIV, hepatitis C and other infections. Injection drug use was verified by asking questions on particular region-specific practices of injection drug use. Persons unable to give a correct answer to the question are not included in the analysis sample [21]. Those who participated in the survey during the last six months were not eligible for participation because of the higher possibility of socially desirable answers, given that they already knew the nature of the questions. This analysis used a sub-sample of the original study participants who reported having a permanent partner who are not IDU (*N=*1379). Instruments for data collection included individual face-to-face, structured interviews (approximately one hour long) and blood testing for HIV and hepatitis C with the use of rapid test kits. A detailed protocol of the survey is described elsewhere [21, 22]. Calculation of the sampled population for each city covered by the survey was based on HIV data in different regions of Ukraine: for cities with high HIV prevalence the sample was 500 respondents; for cities with average levels of the epidemic it was 300–350 respondents; and for cities with low levels the desired sample size was 200–250 respondents. The desired RDS equilibrium was reached in all survey cities, meeting the requirement for conducting RDS [23]. The mean number of waves from each seed was around 10, ranging from 1 (for ineffective seeds) to 24 waves.

The ethical review boards of the L.V. Gromashevsky Institute of Epidemiology and Infectious Diseases and the Sociological Association of Ukraine approved the protocol and materials.

### Measures

The outcome measure was defined as “consistent condom use” if condom use was reported at every event during anal, vaginal, or oral sex with a permanent, casual, or commercial partner during the recall period (90 days) and “inconsistent condom use” if the reported condom use was less than 100% during the recall period. This decision was based on findings from previous research, which showed that consistent condom use is the most effective method of HIV prevention among HIV serodiscordant couples [24, 25].

To identify respondents with a permanent partner, the question “Select an option corresponding to your current marital status” provided the following answer options: “married/live with a woman/ man”, “married but have another sexual partner”, “not officially married, but live with sex partner”.

The primary risk factor of interest was the combination of HIV status and awareness of HIV status. The participants’ HIV status was determined through rapid testing (for blood testing, the study used the NEWVISIONDIAGNOSTICS “PROFITEST” rapid test for antibodies to HIV ½, and the NEWVISIONDIAGNOSTICS “PROFITEST” rapid test for hepatitis C) [22]. Awareness of HIV status was determined through a survey question. Participants were categorized into five groups based on the HIV test conducted during the survey and their reported prior knowledge of HIV status: known HIV+, unknown HIV+, known HIV−, unknown HIV− and undetermined. The undetermined group consisted of those whose awareness about their HIV status could not be ascertained from their answers (they refused to answer about their HIV status or data was missing for this question). Among this category 30% tested positive for HIV and 70% tested negative.

Covariates considered during analyses were selected from analytical domains that previous studies have shown to be associated with risky sexual behaviour, including socio-demographics (e.g., age, education and region), alcohol use during the last 30 days, years of injecting, access to preventive services and self-reported type of main drug used (opiates vs. stimulants). For the sub-sample of the original dataset analyzed here, existing RDS weights were not applicable for several reasons: (1) weighted analyses increase variability; and (2) in studies of association, unweighted analyses with covariates may be preferred to weighted analyses [26]. While the use of RDS weights in multivariable analysis is still in development [27, 28], a similar approach was successfully used for another study utilizing RDS data [29].

### Statistical analyses

Descriptive statistics, such as frequencies, were used to examine the demographic and behavioural characteristics of the sub-sample. Statistically significant association between HIV status knowledge, other covariates and consistent condom use identified during bivariate analysis at *p<*0.05 was used as a background for conducting multivariate analysis.

We performed multivariate analysis on males only, for two reasons: (1) gender differences in risky sexual behaviour among men and women have been discussed previously [5, 30], and (2) male IDUs constituted the majority of our sub-sample. Insufficient statistical power precluded further assessment among females beyond bivariate analyses.

Poisson regression, with robust variance estimates, was utilized to identify associations between knowledge of HIV status and consistent condom use while adjusting for other factors pertinent to injection drug using males who reported having non-injection drug using partners. The multivariate model also included factors widely discussed in the literature as predictors of consistent condom use: age, education, being a client of an NGO, years of injection, primary drug and region. We chose Poisson regression because the prevalence of the outcome is high, resulting in artificially large odds ratios and imprecise variance estimates. Such an approach has been justified in previous studies [31–33].

The primary goal during multivariate analysis was to choose the most parsimonious model which addresses our research question, namely whether there is an association between the HIV status awareness and sexual behaviour. In doing so, we have included all the variables significant at *p<*0.05 level during bivariate analysis as well as important variables identified from the literature. The best fit model was selected using a backwards method removing factors that were not associated at a *p<*0.05 level of significance. Factors removed were assessed for confounding by adding each back into the model to assess the impact on the exposure-outcome association.

Possible interactions were checked and reported if significant. Missing data are mentioned in the tables but were not included in the bivariate and multivariate analyses.

Model fit was assessed using a Chi-square goodness of fit test. Additionally, for each covariate pattern a comparison of observed proportions and model-based predicted proportions were checked to identify groups for whom the model did not fit well.

Statistical analysis was conducted using STATA version 12.0 (College Station, TX).

## Results

Table 1 provides the demographic data on the study sample of the 1,379 active IDUs who reported that their permanent partners had never injected drugs. Most were men (92.3%) and approximately 90% of the sample were 25 years and older with at least a secondary education (nine years or more). The distribution of employment status differed between the genders, with females tending to report being unemployed more than males (*p*<0.01).

**Table 1:** Descriptive statistics of study subjects by gender^a^^,^^b^

	Male		Female			Total	
				
Characteristic	*N*	%	*N*	%	*p*-value	*N*	%
	1279	92.7	100	7.3		1379	
Mean age	34		35		<0.001	34	
Occupation							
Student	14	1.1	2	2.0	<0.001	16	1.2
Permanently employed	430	33.6	31	31.0		461	33.4
Occasional earnings	568	44.4	22	22.0		590	42.8
Unemployed	210	16.4	24	24.0		234	17
Homemaker	11	0.9	15	15.0		26	1.9
Disabled	40	3.1	5	5.0		45	3.3
Retired	5	0.4	1^c^	1.0		6	0.2
Missing	1	0.1				1	0.1
Education							
Primary	143	11.2	18	18.0	0.06	161	11.7
Secondary	712	55.8	46	46.0		758	55
Higher, technical college	422	33.1	36	36.0		458	33.3
Missing^d^	2	0.2				2	0.2
Age of first sexual contact (y)							
< 15	386	30.2	12	12.0	<0.001	398	28.9
15	241	18.8	11	11.0		252	18.3
16	334	26.1	24	24.0		358	26
17–18	247	19.3	41	41.0		288	20.9
19 +	63	4.9	12	12.0		75	5.4
Difficult to answer, do not remember	8	1	0			3	0.2
Total No. of sexual partners during past three months							
0 partners	2	0.2	0	–	0.17	2	0.2
1 partner	993	77.6	88	88.0		1 081	78.4
2–3 partners	156	12.2	5	5.0		161	11.7
4 and more	98	7.9	5	5.0		103	7.7
Question was not asked	30	2.4	2	2.0		32	2.3
Have ever had an HIV test before							
Yes	809	63.3	71	71.0	0.27	880	63.8
No	466	36.4	29	29.0		495	35.9
No answer	4	0.3				4	0.3
Result of HIV test within survey							
HIV +	218	17.0	22	22.0	0.21	240	17.4
HIV −	1 061	83.0	78	78.0		1 139	82.6
Result of HCV test within survey							
HCV +	464	36.3	35	35.0	0.8	499	36.2
HCV −	815	63.7	65	65.0		880	63.8
Condom use with permanent partner, 90 days							
Consistent	243	19.0	23	23.0	0.59	266	19.3
Inconsistent	958	74.9	70	70.0		1 028	74.6
Difficult to answer/do not remember	22	1.72	1	1.0		23	1.7
N/A^e^	56	4.4	6	6.0		62	4.5

a Statistical analysis was conducted using STATA version 12.0

b individuals who reported having a permanent partner who do not inject drugs and have not injected them in the past (*N =*1379); selected socio-demographic characteristic are shown

c maternity leave

d missing data are mentioned in the tables and were not computed for the bivariate or multivariate analyses

e was not sexually active during the recall period.

The majority (63.8%) of the sample (*N=*880) had been previously tested for HIV; this proportion was higher among women (71%, *N=*71). Overall, 17.4% reported testing HIV-positive (*N=*240) and 36.2% reported being HCV-positive (*N=*499) (Table 1).

One-third of the sample reported the first sexual contact was before 15 years of age; more than 90% of respondents reported having had their first sexual contact by age 19. The majority of the IDUs (almost 80%) reported having only one sexual partner during the last 90 days and 19% of the sample reported consistent condom use with their permanent partner during the same recall period.

Reported consistent condom use varied between 15.5% (unknown HIV−, *N=*61) and 37.5% (known HIV+, *N=*27), (Table 2); the sample average was 19.3% (Table 1). Our results show (Table 2) that males who are aware of their HIV+ status are more likely to report recent consistent condom use compared to those who are HIV+ and do not know their status (prevalence ratio (PR)=1.56; 95% CI 0.99–2.47). This association remained significant even after adjustment for age, region, education level, years of injection, alcohol use, self-reported primary drug and being an NGO client (PR=1.65 95% CI (1.03−2.64). No association existed between HIV status awareness and reported condom use for those who were HIV− (PR=1.02, 95% CI 0.75−1.35).

**Table 2:** Crude and adjusted prevalence ratios (95% CI) of Poisson regression for association with consistent condom use and one's knowledge of HIV status among male IDUs with permanent partners who are not IDU, Ukraine, 2011

Knowledge of HIV status	*N* total	*N* of consistent condom use	%	Crude PR	95% CI	Adjusted PR	95% CI
HIV+ unknown	100	24	24.0	1.00		1.00	
HIV+ known	72	27	37.5	**1.56**	**0.99–2.47**	**1.65**	**1.03–2.64**
HIV− unknown	394	61	15.5	1.00		1.00	
HIV− known	533	84	15.8	1.02	0.75–1.38	0.98	0.72–1.33
Not determined	107	26	24.3	**1.57**	**1.05–2.36**	**1.58**	**1.05–2.40**

The bold values are significant at p<0.05 level.

Region of residence and frequency of alcohol use during the last 30 days also were found to be associated with consistent condom use (data not shown), however, they did not have a major confounding effect on the association between consistent condom use and HIV status awareness.

Because over 90% of the sample was male, the adjusted PRs for the model which included both males and females were of the same direction and magnitude as the models presented for males alone (PR=1.61; CI 1.02−2.54 for HIV+ unaware compared to HIV+ aware and PR=1.01; 95% CI 0.74−1.37 for HIV− aware compared to HIV− unaware).

## Discussion

The potential for the HIV epidemic to bridge from the primary reservoir of IDUs to the general population in Ukraine is real and concerning. Our study shows that the level of consistent condom use is still low among IDUs who are HIV+: only about one in three HIV+ injection drug using males who are aware of their status use condoms. Condom use among HIV+ IDUs who do not know their status is even lower.

While the measurement of condom use varies across studies, the findings here substantiate other research in the region. In the most recent survey among Ukrainian commercial sex workers, it was found that only 37% of the sample reported always using a condom with a permanent partner regardless of HIV status [34]. Among the general population, 37.2% of men and 25.2% of women reported using a condom during the last sexual contact according to the most recent survey conducted in Ukraine. The level of use was inversely proportional to the level of intimacy with a partner [35]. In other words, condom use was more common among partners who were in less frequent contact. A study from St. Petersburg also revealed low levels of condom use among potential bridge populations, i.e. persons who have an injection drug using sex partner who personally do not belong to any known risk group. Among those who had a known injection drug using sex partner, 67% reported never using condoms with them, and strikingly, among those with a known HIV+ sex partner 68% reported never using condoms with them [5].

Reported condom use was more common among men who were aware of their HIV+ status. It has been shown in other studies in Ukraine that knowledge of HIV status is associated with less risky sexual behaviour [36–39]. A review of behaviour change and health-related interventions for heterosexual risk reduction among IDUs indicates that known HIV seropositivity greatly increases an IDU's use of condoms to reduce the risk of transmitting HIV to a sex partner(s) [8]. This precautionary behaviour may reflect effective counselling following HIV testing about how to prevent transmission to sexual partners.

Our findings provide supporting data in favour of HIV testing and counselling as a strategy for preventing bridging HIV to the non-injection drug using partners of IDUs in Ukraine. However, this intervention alone is not enough. Recent data on the potential benefit of pre-exposure prophylaxis has already been shown it to be an effective primary HIV-1 prevention strategy among serodiscordant heterosexual couples and indicates it may be a promising tool to mitigate the spread of the epidemic to the general public [19]. Despite valid concerns about the cost-effectiveness of such an approach, containing the epidemic to concentration among high-risk groups is a priority for Ukraine according to recent studies on epidemic transition [3]. Modelling the addition of pre-exposure prophylaxis among different risk groups should be done to assess the costs and potential benefits of adding this prevention strategy in Ukraine and the region. Until such analysis is done, increasing access to ART remains a high priority for public health sector.

Our findings should be viewed within the context of several limitations. First, self-reported sexual behaviour may be prone to socially desirable responses. Data on self-reported STDs among our sample correlates with answers regarding risky sexual behaviour, in particular, those who report consistent condom use also tend to report no STDs, and such information was utilized in previous studies as an outcome measure of risky sexual behaviour [7]. Second, the cross-sectional nature of the data prevents us from making causal inferences about HIV testing and behaviour change. The reasonable explanation is that condom use increased after HIV diagnosis and counselling. In other words, if the cause-effect link went in the opposite direction, we would be forced to accept that condoms increase the risk of acquiring HIV, clearly not a logical conclusion. Third, our data did not provide information on partners’ HIV status, which could greatly influence the decision on condom use among IDUs who are aware of their HIV+ status. Also, our data do not provide the information on any intention to have children, which could be a reason for not using a condom. Even though the intention to have a baby influences a decision to use a condom and, consequently, could influence our results, we believe that the magnitude of such influence is small among this population. Fourth, laboratory confirmation of injection drug use was not feasible. But we sought to minimize false reporting injection drug use by asking specific questions pertinent to the region's typical use behaviours. Finally, the analysis did not address the RDS design. In RDS, individuals are selected with probability proportional to how many friends they have in the target population [40]. Our estimated association between HIV knowledge and condom use could be affected by the RDS sampling technique only if important effect modifiers exist and were not taken into account in the analysis.

## Conclusions

Our results reinforce the importance of HIV counselling and testing as an intervention tool, but this strategy is not enough to control the HIV epidemic in Ukraine. Prevention could also be provided by antiretroviral therapy and behavioural interventions for people aware that they are infected. Nevertheless, when there is high potential for epidemic spread outside the at-risk group, innovative and proven strategies, such as pre-exposure prophylaxis for non-injecting sex partners of IDUs, should be assessed as a part of a complete prevention package.
